# Development and relative validation of a food frequency questionnaire for French-Canadian adolescent and young adult survivors of acute lymphoblastic leukemia

**DOI:** 10.1186/s12937-018-0355-9

**Published:** 2018-04-21

**Authors:** Sophia Morel, Olivia Portolese, Yasmine Chertouk, Jade Leahy, Laurence Bertout, Caroline Laverdière, Maja Krajinovic, Daniel Sinnett, Emile Levy, Valérie Marcil

**Affiliations:** 1 Research Centre, Sainte-Justine University Health Center, Department of Nutrition, Université de Montréal, Montreal, QC H3T 1C5 Canada; 2 Research Centre, Sainte-Justine University Health Center, Department of Pediatrics, Université de Montréal, Montreal, QC H3T 1C5 Canada; 3 Institute of Nutrition and Functional Foods, Laval University, Quebec City, QC G1V 0A6 Canada

**Keywords:** Dietary assessment, Food frequency questionnaire, Dietary intake, Adolescent, Young adults, Quebec

## Abstract

**Background:**

Survivors of childhood acute lymphoblastic leukemia (cALL) experience cardiometabolic and bone complications after treatments. This study aimed at developing and validating an interview-administrated food frequency questionnaire (FFQ) that will serve to estimate the impact of nutrition in the development of long-term sequalea of French-Canadian cALL survivors.

**Methods:**

The FFQ was developed to assess habitual diet, Mediterranean diet score, nutrients promoting bone health and antioxidants. It was validated using a 3-day food record (3-DFR) in 80 cALL survivors (50% male) aged between 11.4 and 40.1 years (median of 18.0 years). Reproducibility was evaluated by comparing FFQs from visit 1 and 2 in 29 cALL survivors.

**Results:**

When compared to 3-DFR, the mean values for macro- and micronutrient intake were overestimated by our FFQ with the exception of lipid-related nutrients. Correlations between nutrient intakes derived from the FFQs and the 3-DFRs showed moderate to very good correlations (0.46–0.74). Intraclass correlation coefficients assessing FFQ reproducibility ranged from 0.62 to 0.92, indicating moderate to good reliability. Furthermore, classification into quartiles showed more than 75% of macro- and micronutrients derived from FFQs 1 and 2 classified into the same or adjacent quartile.

**Conclusions:**

Overall, our results support the reproducibility and accuracy of the developed FFQ to appropriately classify individuals according to their dietary intake. This validated tool will be valuable for future studies analyzing the impact of nutrition on cardiometabolic and bone complications in French-speaking populations.

**Electronic supplementary material:**

The online version of this article (10.1186/s12937-018-0355-9) contains supplementary material, which is available to authorized users.

## Background

Acute lymphoblastic leukemia (ALL) accounts for approximately one fourth of all childhood malignancies [1]. Fortunately, cure rates now exceed 80%, which allows a growing number of childhood survivors to live into adulthood [1]. However, survivors may face severe long-term sequelae years after the end of treatments [2, 3]. In particular, studies on childhood ALL (cALL) survivors have reported a high prevalence of the typical components of the metabolic syndrome (MetS), namely obesity [4], hypertension [5], glucose intolerance [6] and dyslipidemia [7]. Furthermore, cALL treatments can affect bone growth and development leading to musculoskeletal morbidities that can compromise bone health [8]. Inadequate nutrition has been associated with an increased risk of developing long-term sequelae in cALL survivors [9–11], while the inclusion of specific nutrients or dietary patterns was found protective [10, 11]. Key nutrients for bone health include proteins, calcium and vitamin D [12]. In addition to their anti-inflammatory actions [13, 14], antioxidants (polyphenols, vitamins and minerals: e.g. vitamins A, C, E and selenium) mainly found in plant foods and whole grains can improve blood pressure and lipid profile [14–16]. Food scores have been developed to evaluate the adherence to Mediterranean diet and are mainly used in nutritional epidemiologic studies [17]. This diet is characterized by high intake of fruits, vegetables, legumes, fish, whole grains, nuts, and olive oil along with moderate consumption of dairy products and wine, as well as low intake of red and processed meats and foods that contain high amounts of added sugars [18]. The cardio protective properties of a Mediterranean dietary pattern have been widely reviewed [18–20].

Food frequency questionnaires (FFQs) and food records are tools that can be used to estimate individual food intake. Both tools have limitations and are subjected to measurement error and bias. Quantifying precise food intakes with FFQs require challenging respondents with complex and difficult cognitive tasks (e.g., recall and abstraction/estimation of averages over time) [21]. Also, because FFQs are limited by food list content, they need to be adapted to the study objectives and specific populations. On the other hand, self-reported food records might be incomplete or lack details regarding quantities or types of foods consumed. A participant may modify his diet to simplify the task or may report what is, in his perspective, an ideal diet. Poor response rates can also be problematic. Interviewed-administrated FFQs ensure participant’s response but are time-consuming for both the participant and the interviewer. The interviewer can also assist the participant with the task of quantifying intakes. FFQs are common tools to estimate usual food intake and are used in epidemiologic studies to investigate associations between diet and diseases [22].

Currently, there is a great need for a reliable tool to assess the impact of dietary habits in cALL survivors. Therefore, the aim of this work is to develop and validate an interview-administrated FFQ in a population of French-Canadian cALL survivors from the Province of Quebec that assesses habitual diet, Mediterranean diet score, bone health-promoting nutrients, and antioxidants.

## Methods

### Study design

**Fig. 1:**
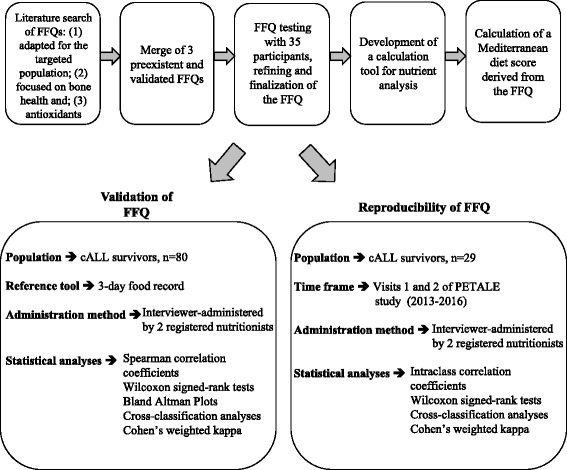
Study design for the development, validation and reproducibility of the FFQ for adolescent and young adult survivors of acute lymphoblastic leukemia living in the Province of Quebec. FFQ: food-frequency questionnaire; cALL: childhood acute lymphoblastic leukemia

The study design includes 3 phases (Fig. 1): the first consists in the 5-step development of the FFQ; the second reflects the validation of the questionnaire in a cohort of 80 participants; and the third tests the reproducibility of the tool by comparing FFQs collected during 2 different visits.

### Study population

Participants enrolled in this study were recruited between January 2013 and December 2016 as part of the PETALE program at Sainte-Justine University Health Center in Montreal [8]. The PETALE study allowed the evaluation of long-term sequelae of cALL survivors, namely cardiotoxicity, cardiometabolic complications, neurocognitive problems, bone morbidity and quality of life issues [8]. Participants were recruited from European-descent cALL survivors living in the Province of Quebec. The PETALE study comprised of two phases. All participants were met in Phase 1 (*n* = 246). Participants identified with extreme phenotypes at Phase 1 (those with best and worst outcomes relative to long-term sequelea) were contacted for additional studies (Phase 2, *n* = 100). Visits of Phases 1 and 2 were, on average, 1 year *(± 21 weeks)* apart. In both phases, the FFQ was administrated to participants on the visit day by registered nutritionists (2 interviewers) and a 3-day food record (3-DFR) was handed out to complete at home. For validation of the FFQ, we included participants who fully completed and returned their 3-DFR. If the 3-DFR was incomplete (e.g., missing day or unspecified quantity of food), the participant was excluded from the validation step. A total of 80 FFQs and the associated 3-DFRs from either phase 1 or 2 were randomly selected for the validation, *n* = 40 for each FFQ interviewer (10 men, 10 boys, 10 women and 10 girls). For assessing FFQ reproducibility, 29 participants who took part in the two phases of the study (visits 1 and 2) and who were interviewed by both nutritionists were selected. The Institutional Review Board of Sainte-Justine University Health Center approved the study and investigations were carried out in accordance with the principles of the Declaration of Helsinki. Written informed consent was obtained from study participants and/or parents/guardians.

### Development of the food frequency questionnaire

The FFQ was derived from 3 validated and published questionnaires. The first one was developed by Goulet et al., was structured to reflect French-Canadians’ dietary habits and was validated among healthy women aged between 30 and 65 years [21]. The second FFQ was developed by Pritchard et al. and targeted nutrients affecting bone health. It was validated in postmenopausal women aged 70.3 ± 4.7 years and mainly Caucasian [23]. The third FFQ was an antioxidant nutrient questionnaire published by Satia et al. in which food items were selected based on the most commonly consumed antioxidant-rich foods. The mean age of participants was 31.9 years, 51% were African American, 52% were female and all were free of chronic disease [24]. The final result consisted of an interviewer-administered FFQ, which comprised 190 items with at least 106 having sub-questions to more accurately describe the nature of the food consumed. The developed FFQ was pre-tested in 35 participants and the tool was then modified accordingly, after addressing issues relating to content and comprehension.

### Administration of the Food Frequency Questionnaire

Two registered nutritionists alternately administered the FFQ to participants using measuring cups to facilitate portion size estimation. Importantly, a portion size was specified for each food item. The standard portion size was adjustable by using fractions in the frequency column. An extra column was also available to indicate any other unit of measurement. Quantities were converted when the FFQ was analyzed. Participants were surveyed regarding the frequency each item was consumed during the past 2 months on a daily, weekly or monthly basis. Supplementary open- and closed-ended questions were added to support and complete participants’ reported food intake by addressing usual foods that might have been forgotten, recent modifications in eating habits, weekly frequency of the main food groups, as well as use, dosage and frequency of dietary supplements.

### 3-day food record

At each visit (Phases 1 and 2), instructions were given to participants to complete a 3-DFR at home in the following weeks. Participants were asked to report their food and beverage intake for 1 day over the weekend and 2 days of the week. They were encouraged to choose days that reflected their usual diet and to not restrict their food intake. Emphasis was placed on providing comprehensive information relative to the portion size. While food did not have to be weighed, participants were encouraged to use measuring cups and spoons to estimate portions. A section for dietary supplements gathered information on dosage, frequency and brand.

### Nutrient calculation tools

#### Food frequency questionnaire

To evaluate nutrient intakes derived from the FFQs, a nutrient calculation tool was built in-house using Microsoft® Excel® 2011. Nutrient values were obtained from the 2010 Canadian Nutrient File (CNF) that includes about 5500 food items commonly consumed in Canada [25]. If a food item was not found in the CNF, we used the United States Department of Agriculture National Nutrient Database for Standard Reference release 25, the major source of food composition data in the United States [26]. When necessary, we contacted companies to obtain nutrient values of their product. Energy and nutrient intakes were compiled, particularly proteins, carbohydrates (CHO), lipids, saturated fatty acids (SFA), monounsaturated fatty acids (MUFA), polyunsaturated fatty acids (PUFA), cholesterol, dietary fiber, alcohol, calcium, iron, folate, and vitamins A, C, D and E.

#### 3-day food record

The 3-DFRs were analysed with the application Nutrific® developed by the Department of Food Science and Nutrition, Université Laval (https://nutrific.fsaa.ulaval.ca). Nutrient values from this application are derived from the 2010 CNF and, when a specific food item was not available, its nutrient value was added to the database.

### Mediterranean diet score

Using the FFQ, we calculated a Mediterranean diet score based on a validated short questionnaire developed by Martinez-Gonzalez et al. [27]. The questionnaire is composed of nine food groups and assesses the consumption of cardio protective elements included in the Mediterranean diet. We calculated the Mediterranean diet score using the FFQ by regrouping each food item corresponding to each category (Table 1).

**Table 1:** Calculation of the Mediterranean diet score

	Points if yes
Olive oil (≥1 spoon/day)	+1
Fruits (≥1 serving/day)	+1
Vegetables of salad (≥1 serving/day)	+1
Fruits (≥1 serving/day) *and* Vegetables or salad (≥1 serving/day)^a^	+1
Legumes (≥ 2 servings/week)	+1
Fish (≥ 3 servings/week)	+1
Wine (≥1 glass/day)	+1
Meat (< 1 serving/day)	+1
[White bread (< 1 slice/day) *and* rice (< 1 cup/week)] *or* whole-grain bread (> 5 slices/week)^b^	+ 1

^a^One point is added when ≥1 serving/day of both fruits and vegetables is consumed

^b^One point is added when either consumption of both white bread and rice is low or when consumption of whole-grain bread is high. Adapted from Martinez-Gonzalez et al. 2004. Development of a short dietary intake questionnaire for the quantitative estimation of adherence to a cardioprotective Mediterranean diet. European Journal of Clinical Nutrition 58: 1550–1552

### Statistical analysis

#### Relative validity

Medians and interquartile ranges were calculated for energy and nutrient intakes for the FFQ and the 3-DFR. Wilcoxon signed-rank tests were used to compare values obtained from both tools, as the data were not normally distributed. We used Spearman correlations to assess the relationships for energy, nutrient intakes and energy-adjusted nutrient intakes. The residual method proposed by Willett et al. was used to calculate the energy-adjusted variables [28]. With this method, the nutrient intakes of each individual are regressed on their total energy intakes. The residual from the regression represent the differences between each individual’s actual intake and the intake predicted by their total energy intake [28]. The following classification was utilized to interpret correlation coefficients: very well correlated (coefficient 0.7 to 0.9), well correlated (coefficient 0.5 to 0.7) [29] and moderately well correlated (coefficient 0.3 to 0.5). This classification was also used to interpret the correlation coefficients in one of the FFQ that was used to develop our questionnaire [21]. Bland Altman Plots were used to assess agreement between the two tools for intakes of energy, macronutrients and micronutrients (vitamins A, C, E and D, and calcium). The limits of agreements were calculated by using the mean and the standard deviation (SD) of the differences between the two measurements (mean difference ± 1.96 * SD) [12, 30–32]. Through a graph representation, the differences of the two-paired measurements were plotted against the mean of the two measurements [32].

Cross-classification analyses were completed to validate agreement between the two tools in terms of proportions of participants’ energy and nutrient intakes, classified into the same or contiguous quartiles (same ±1 quartile) or in opposite quartile (misclassified). Crude values of nutrient intakes were used for these analyses. Cohen’s quadratic weighted kappa values were calculated. To interpret the kappa values, we used the arbitrary cut-off points proposed by Flight et al. and Landis and Koch [33, 34]: less than 0.20 indicated poor agreement, 0.21–0.40 fair agreement, 0.41–0.60 moderate agreement, 0.61–0.80 good agreement, and 0.81–1.00 very good agreement.

#### Reproducibility

Medians and interquartile ranges were calculated for energy and nutrient intakes for the FFQs at visits 1 and 2. Wilcoxon signed-rank tests were also used to compare values obtained from both visits. To measure the FFQ reproducibility, we calculated intraclass correlation coefficients between crude values obtained at visits 1 and 2 using the two-way mixed model and computed the absolute agreement type [35]. Energy-adjusted values were not used to calculate intraclass correlation coefficients because, due to the small sample size (*n* = 29), any modification in the composition of diet would greatly impact the residual values and the correlation coefficients. Data on nutrient intake were log-transformed because they were not normally distributed. To assess the quality of reliability, we used the scale suggested by Koo et al. for intraclass correlation coefficients: less than 0.5 indicated poor reliability, 0.5 to 0.75 moderate, 0.75 to 0.9 good, and greater than 0.90 excellent reliability [35].

Intakes of energy, proteins, CHO, lipids, calcium, vitamins A, C, D, E and Mediterranean diet score derived from the FFQs at visits 1 and 2 were also classified into quartiles and cross-classification analyses were completed to validate agreement between the two FFQs. Crude values not adjusted for energy intake were used. Cohen’s quadratic weighted kappa values were also calculated to measure the agreement between the two FFQs.

Statistical analyses were performed with IBM SPSS Statistics Version 24, and the extension bundle Stats weighted kappa.spe for SPSS was used.

## Results

Descriptive characteristics of participants are summarized in Table 2. Each registered nutritionist interviewed 40 participants. All participants were Caucasian and French-speaking.

**Table 2:** Descriptive characteristic of participants

	Total *N* = 80	Adults *N* = 40	Children *N* = 40
Gender, *N* (%)			
Male	80 (50.0)	40 (50.0)	40 (50.0)
Age at visit, years			
Mean (SD)	21.9 (7.2)	27.8 (5.6)	16.0 (1.5)
Median (range)	18.0 (11.4–40.1)	26.7 (18.2–40.1)	16.2 (11.4–17.9)

Subjects were stratified according to age (adults: ≥ 18 years old and children: < 18 years old)

### Relative validity

The response rate obtained was 75 and 45% for the 3-DFR in Phases 1 and 2, respectively. Therefore, on a total of 100 participants who were invited to both phases, 39 completed and handed two 3-DFRs. We evaluated the time difference between the administration of the FFQ and the completion of the 3-DFR. We found that 66 of 80 participants (82.5%) filled out the 3-DFR within a month following the administration of the FFQ. Four of 80 participants (5%) completed the food record after 1.5 to 3.5 months and 10 of 80 (12.5%) did not inscribe the date.

**Fig. 2:**
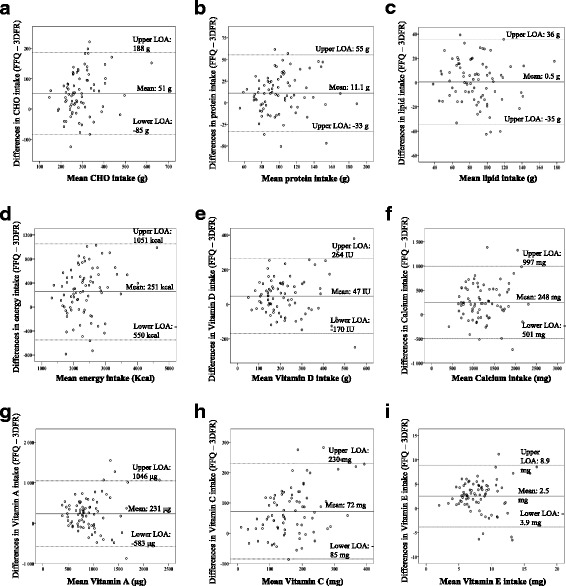
Bland-Altman plot showing agreement between the average FFQ and the average 3-DFR for **a** carbohydrates, **b** proteins, **c** lipids, **d** energy intake, **e** vitamin D, **f** calcium, **g** vitamin A, **h** vitamin C, **i** and vitamin E. A mean of 0 indicates that the 2 methods are in perfect agreement. LOA, limit of agreement; IU, international unit

The median values of daily energy and nutrient intakes derived from the FFQ and the 3-DFR are reported in Table 3. Two of 3 macronutrients derived from the FFQ showed higher values compared to the 3-DFR (proteins + 12.7% and CHO + 20.0%), resulting in higher reported energy intake (+ 12.1%). Reported intakes of dietary fiber and micronutrients (vitamins A, C, E and D, calcium, iron and folate) were also higher with the FFQ, differences ranging between + 16.0% (iron) and + 52.6% (vitamin C). Similar trends were observed when children and adults were analyzed separately (Additional file 1: Tables S1 and S2). The variability of differences between the FFQ and the 3-DFR are illustrated in the Bland Altman plots (Fig. 2) for the intakes in energy, CHO, proteins, lipids, calcium and vitamins D, C, A and E.

**Table 3:** Food frequency questionnaires and 3-day food records: Comparison of nutrient median (inter-quartile range) daily intake and correlation coefficients

	Median (IQR) daily intake			Correlations^c^	
	3-DFR	FFQ^b^	Difference % (IQR)^a^	Unadjusted	Energy-adjusted^d^
Energy (kcal)	2093 (1860–2518)	2439 (2020–2802)^***^	12.1 (−1.1–24.9)	0.72^**^	
Protein (g)	87.2 (78.4–106)	102 (82.9–122)^***^	12.7 (−3.2–32.0)	0.59^**^	0.47^**^
CHO (g)	248 (214–307)	295 (254–379)^***^	20.0 (1.75–43.0)	0.50^**^	0.39^**^
Lipids (g)	82.8 (68.3–103)	84.1 (74.8–103)	2.5 (− 12.9–16.5)	0.74^**^	0.28^*^
SFA (g)	28.9 (21.8–37.3)	28.3 (23.7–32.7)	−4.7 (− 17.9–18.7)	0.69^**^	0.33^**^
MUFA (g)	29.2 (23.1–35.6)	29.8 (25.8–36.8)	2,6 (−14.2–25.9)	0.61^**^	0.25^*^
PUFA (g)	15.0 (10.7–19.5)	15.8 (12.8–19.3)	3.2 (− 10.8–26.1)	0.58^**^	0.33^**^
Cholesterol (mg)	266 (195–351)	256 (197–332)	− 9.1 (− 31.1–32.0)	0.46^**^	0.24^*^
Dietary fiber (g)	17.3 (13.6–21.0)	23.8 (18.1–29.8)^***^	31.1 (0.4–60.0)	0.50^**^	0.58^**^
Alcohol (g)	0.0 (0.0–4.6)	1.1 (0.0–9.5)^*^	–	0.59^**^	0.47^**^
Vitamin A (μg)	720 (564–988)	950 (747–1171)^***^	34.3 (− 8.5–80.4)	0.47^**^	0.41^**^
Vitamin C (mg)	122 (74.9–183.2)	187 (139–257)^***^	52.6 (14.5–122)	0.55^**^	0.50^**^
Vitamin E (mg)	6.2 (4.6–8.4)	8.9 (7.35–10.6)^***^	50.6 (16.4–73.5)	0.54^**^	0.47^**^
Vitamin D (IU)	176 (114–255)	226 (157–296)^**^	24.3 (− 16.0–83.1)	0.58^**^	0.50^**^
Calcium (mg)	1080 (690–1407)	1307 (1002–1654)^***^	23.0 (2.5–49.6)	0.70^**^	0.52^**^
Iron (mg)	12.5 (11.0–16.3)	16.0 (13.2–19.0)^***^	16.0 (3.7–45.6)	0.49^**^	0.42^**^
Folate (mcg)	347 (296–437)	492 (388–620)^***^	39.6 (14.4–72.4)	0.46^**^	0.41^**^

Values are median (IQR), *N* = 80

*IQR* inter-quartile range, *3-DFR* 3-day food record, *FFQ* food frequency questionnaire, *CHO* carbohydrates, *SFA* saturated fatty acids; *MUFA* monounsaturated fatty acids, *PUFA* polyunsaturated fatty acids

Note: Data presented as means ± SD are available in Additional file 1: Table S9

^*^P ≤ 0.05; ^**^P ≤ 0.01; ^***^P ≤ 0.0001

^a^(Daily intake as per FFQ – Daily intake as per 3-DFR)/(Daily intake as per 3-DFR) × 100

^b^Wilcoxon signed-rank tests were used to compare values obtained from both FFQ and 3-DFR

^c^Spearman correlations were used to assess the relationship of energy, nutrient intakes and energy-adjusted nutrient intakes between 3-DFR and FFQ

^d^The residual method was used to calculate the energy-adjusted variables

Spearman correlations between nutrient intakes derived from the FFQs and the 3-DFRs are shown in Table 3. Crude analyses revealed very good correlations for lipids and good correlations for proteins, SFA, MUFA, PUFA, alcohol, vitamins C, D and E, and calcium. CHO, cholesterol, dietary fiber, vitamin A, iron and folate were moderately well correlated. After adjustment for energy intake, the quality of correlations remained unchanged for vitamins A C, D and E, calcium, iron and folate. A slight reduction in the coefficient for proteins and CHO adjusted for energy was observed. Adjusting for energy decreased the coefficients for all lipids: total lipids (0.28 vs. 0.74), SFA (0.33 vs. 0.69), MUFA (0.25 vs. 0.61), PUFA (0.33 vs. 0.58) and cholesterol (0.24 vs. 0.46). No major differences in correlation coefficients were noticeable when children and adults were analysed separately (Additional file 1: Tables S1 and S2). However, when the analyses were performed separately for males and females, the correlation coefficients followed the same trend with the exception of the female children group (girls) in which coefficient correlations were lower. The nutrients for which we found lower coefficients in female group were energy, proteins, CHO, cholesterol, vitamin C and iron, coefficients ranging between 0.07 (cholesterol) and 0.48 (energy intake) (Additional file 1: Tables S3, S4, S5, S6, S7 and S8).

Bland-Altman plots show that the FFQ overestimated energy intake by an average of 251 kcal/day, CHO intake by 51 g/day and protein intake by 11 g/day (Fig. 2). Micronutrients were also overestimated by the FFQ (vitamin A: 231 μg, vitamin C: 72 mg, vitamin E: 2.5 mg, vitamin D: 47 IU and calcium: 248 mg) (Fig. 2). More than 93% of the points fell within the limits of agreement for energy intake, CHO, proteins, lipids, vitamin A, C, E and D, and calcium.

Over 82% of participants’ energy intake, macronutrients- and lipid-related nutrients derived from FFQ and 3-DFR were classified into the same or adjacent quartile (Table 4). Weighted kappa values were above 0.41 for all nutrients indicating moderate to good agreement with the exception of iron (0.37, fair agreement).

**Table 4:** Food frequency questionnaires and 3-day food records: cross-classification analysis of energy and nutrient intakes

	% Classified into same quartile^1^	% Classified into same ±1 quartile^a^	% Misclassified^b^	Weighted^b^ Kappa
Energy	47.5	40.0	12.5	0.64
Protein	45.0	37.5	17.5	0.52
CHO	45.0	32.5	22.5	0.46
Lipids	51.3	40.0	8.7	0.70
SFA	50.0	36.3	13.7	0.63
MUFA	35.0	51.3	13.7	0.55
PUFA	48.7	35.0	16.3	0.58
Cholesterol	30.0	51.3	18.7	0.42
Dietary fiber	46.3	30.0	23.7	0.43
Alcohol	57.5	28.7	13.8	0.67
Vitamin A	41.3	33.7	25.0	0.44
Vitamin C	38.7	43.8	17.5	0.47
Vitamin E	41.3	40.0	18.7	0.47
Vitamin D	40.0	41.3	18.7	0.51
Calcium	53.7	33.8	12.5	0.64
Iron	40.0	36.3	23.7	0.37
Folate	37.5	43.7	18.8	0.45

Values are expressed in percentage, *N* = 80

*3-DFR* 3-day food record, *FFQ* food frequency questionnaire, *CHO* carbohydrates, *SFA* saturated fatty acids, *MUFA* monounsaturated fatty acids, *PUFA* polyunsaturated fatty acids

^a^Cross-classification analyses were completed to validate agreement between FFQ and 3-DFR in terms of proportions of participants classified into the same or same ±1 quartile or misclassified

^b^Weighted kappa was used to measure the agreement between FFQ and 3-DFR. Analyses were completed on crude values not adjusted for energy intake

Intraclass correlations assessing FFQ reproducibility between visits 1 and 2 are shown in Table 5. Coefficients for macronutrients ranged between 0.76 (lipids) and 0.81 (CHO), indicating good reliability, which translated into a 0.84 coefficient for energy intake demonstrating good reliability. For micronutrients, intraclass correlation coefficients ranged from 0.68 (vitamin A) to 0.88 (vitamin D) with the indication of moderate to good reliability. Coefficients were found lower for three nutrients: MUFA (0.62), PUFA (0.56) and dietary fiber (0.63), but still indicating moderate reliability. Classification into quartiles showed that more than 75% of macro- and micronutrients derived from FFQs visit 1 and visit 2 were classified into the same or adjacent quartile (Table 6). Notably, 86% of Mediterranean diet scores were classified into the same or adjacent quartile. Weighted kappa values varied from 0.52 (lipid) to 0.66 (CHO) for energy intake and macronutrients and from 0.46 (vitamin C) to 0.66 (vitamin D) for micronutrients, indicating moderate to good agreement for both groups. Weighted kappa value coefficient for the Mediterranean score was 0.68 indicating good agreement. No statistical differences were found between the mean values of daily energy and nutrient intakes derived from the FFQs from both visits. The variability between FFQs visit 1 and 2 of the differences for energy, CHO, protein and lipids are shown in Bland Altman plots (Additional file 2: Figure S1).

**Table 5:** Food frequency questionnaires at visits 1 and 2: Comparison of nutrient median (inter-quartile range) daily intake and intraclass correlation coefficients

	Median (IQR) daily intake			
	FFQ V1	FFQ V2^b^	Difference % (IQR)^a^	Correlations^c^
Energy (kcal)	2533 (2036–2892)	2416 (1941–3062)	4.9 (−16.6–18.7)	0.84^***^
Protein (g)	106 (92.3–127)	100 (88.4–140)	4.8 (− 15.9–12.8)	0.79^***^
CHO (g)	308 (254–380)	296 (226–368)	4.9 (− 10.9–24.7)	0.81^***^
Lipids (g)	91 (67.2–103)	85.7 (68.7–114)	−7.0 (− 15.4–13.0)	0.76^***^
SFA (g)	28.0 (19.0–31.6)	26.8 (22.8–37.0)	−4.3 (− 21.9–15.3)	0.81^***^
MUFA (g)	30.3 (24.3–39.0)	30.9 (24.4–43.5)	−6.8 (− 17.9–18.2)	0.62^**^
PUFA (g)	16.5 (12.2–20.1)	18.1 (13.9–21.9)	−12.4 (− 29.2–1.2)	0.56^*^
Cholesterol (mg)	265 (201–334)	319 (210–423)	−7.4 (− 24.2–6.7)	0.83^***^
Dietary fiber (g)	23.8 (15.9–29.9)	24.2 (16.2–31.4)	−4.4 (− 22.6–16.4)	0.63^**^
Alcohol (g)	2.8 (1.1–12.4)	3.3 (1.5–12.7)	− 6.2 (− 29.0–4.5)	0.92^***^
Vitamin A (μg)	904 (737–1158)	974 (775–1357)	−8.5 (− 19.3–15.0)	0.68^**^
Vitamin C (mg)	191 (131–254)	178 (121–214)	− 6.0 (− 16.1–19.9)	0.83^***^
Vitamin E (mg)	8.8 (6.7–12.9)	8.8 (6.9–12.7)	4.0 (−21.2–20.6)	0.86^***^
Vitamin D (IU)	224 (168–260)	237 (191–281)	−0.6 (− 13.4–18.6)	0.88^***^
Calcium (mg)	1220 (1045–1627)	1240 (1106–1552)	−3.6 (− 15.2–12.5)	0.87^***^
Iron (mg)	15.6 (13.7–19.1)	15.3 (12.8–19.1)	− 0.9 (− 14.0–19.6)	0.76^***^
Folate (mcg)	452 (398–684)	507 (387–664)	0.0 (−22.4–12.0)	0.76^***^

Values are median (IQR), *N* = 29

*IQR* inter-quartile range, *FFQ* food frequency questionnaire, *CHO* carbohydrates, *SFA* saturated fatty acids, *MUFA* monounsaturated fatty acids, *PUFA* polyunsaturated fatty acids

Note: Data presented as means ± SD are available in Additional file 1: Table S10

^*^P ≤ 0.05; ^**^P ≤ 0.01; ^***^P ≤ 0.0001. V1, visit 1; V2, visit 2

^a^(Daily intake as per FFQ V1 – Daily intake as per FFQ V2)/(Daily intake as per FFQ V2) × 100

^b^Wilcoxon signed-rank tests were used to compare values obtained from both FFQ V1 and FFQ V2. No statistical differences were found

^c^Intraclass correlations between FFQ V1 and FFQ V2 based on log transformed values. Analyses were completed on crude values not adjusted for energy intake

**Table 6:** Food frequency questionnaires visit 1 and 2: cross-classification analysis of energy intake, nutrient intake and Mediterranean diet score

	% Classified into same quartile^a^	% Classified into same ±1 quartile^a^	% Misclassified^a^	Weighted Kappa^b^
Energy	44.8	41.4	13.8	0.60
Protein	37.9	48.3	13.8	0.57
CHO	48.3	41.4	10.3	0.66
Lipids	44.8	34.5	20.7	0.52
Vitamin A	48.3	27.6	24.1	0.49
Vitamin C	44.8	41.4	13.8	0.46
Vitamin E	51.7	31.0	17.3	0.52
Vitamin D	51.7	37.9	10.4	0.66
Calcium	51.7	34.5	13.8	0.63
Mediterranean diet score	58.6	27.6	13.8	0.68

Values are expressed in percentage, *N* = 29

^a^Cross-classification analyses were completed to validate agreement between the two FFQs in terms of proportions of participants classified into the same or same ±1 quartile or misclassified

^b^Weighted kappa was used to measure the agreement between the two FFQs. Analyses were completed on crude values not adjusted for energy intake. CHO: carbohydrates

## Discussion

We have developed and relatively validated an interview-administrated FFQ specific for the cALL survivors from the Province of Quebec, Canada to assess habitual diet, Mediterranean score and nutrients promoting bone health and antioxidant defense.

We found good correlation coefficients between our FFQ and the 3-DFR for most of the nutrients. As previously stated, correlation coefficients are generally higher for interviewer–administered than for self-administered FFQs [36, 37]. The precise and comprehensive food list of our questionnaire and its adaptation to the population’s dietary habits contributed to its good accuracy. Also, to limit bias based on seasonal variations of diets [38], our FFQ interrogated food consumption in the past 2 months.

Energy adjusted nutrient intakes were calculated for both tools to reduce the confounding impact of the total energy intake on specific nutrients. We found that, for CHO, proteins and micronutrients, correlations were slightly impacted by total energy intake, while lipids and lipid-related nutrient correlation coefficients were significantly decreased. The reduction of the correlation coefficient of the energy intake adjusted lipids reflects the variation in lipid consumption independently of total energy intake between the 3-DFR and the FFQ. Thereby, the contribution of lipids to total energy intake was lower in the FFQ compared to the 3-DFR. The correlation coefficients found between energy-adjusted nutrients derived from FFQs and 3-DFRs were similar to those reported in a literature review (range between 0.4 and 0.7) [39], with the exception of lipids and lipid-related nutrients for which coefficients were lower in our study.

Compared to 3-DFR, mean values for macro- and micronutrient intakes were overestimated by our FFQ with the exception of lipid-related nutrients. The differences were lower than 20% for all macronutrients. These results concur with many FFQ validation studies that have reported overestimations for energy and nutrient intakes compared to food records or 24-h recalls [30, 31, 40–48]. Still, other groups reported underestimation of energy and nutrient intakes [21, 37, 49, 50]. To our knowledge, only one other study aimed to validate a FFQ specifically for childhood cancer survivors [49]. Conversely to our findings, their FFQ revealed substantial underreporting of dietary intakes compared to repeated 24-h diet recalls. However, this comparison is flawed by the study smaller sample size (only 16 participants aged between 5 and 22 years), the different populations (Americans versus French Canadians) and inclusion criteria as well as the use of 24-h diet recalls as a reference tool [49].

We found larger differences between the FFQs and the 3-DFRs in micronutrients than in macronutrients, the latter being found in a wider range of food. Hence, if foods containing specific micronutrients were not consumed during the days covered by the 3-DFR, this will likely result in a poor correlation with the FFQ. Moreover, the detailed list of fruits and vegetables in the FFQ could explain the observed overestimations for vitamin A, vitamin C and dietary fiber. Of note, in the Province of Quebec, seasons have a considerable impact on the availability, variety and financial accessibility of several fruits and vegetables. Thereby, any delay in completing the 3-DFR could have impacted the reported consumption of fruits/vegetables and the above-mentioned nutrients. However, in our study, most of the 3-DFR were filled out and returned within a month following the completion of the FFQ. Consequently, seasons probably did not influence the observed differences in micronutrients.

For our analyses, we used correlations in conjunction with the Bland-Altman method. While correlations quantify the degree to which two variables are related at an individual level [51], a high correlation does not automatically imply that there is good agreement between the two methods [32]. Bland-Altman plot is a method to assess the agreement between two quantitative measurements by constructing limits of agreement [32]. The plot illustrates the differences between the tools against the mean of the two tools. The closer the mean of differences is to zero and the narrower agreement interval is, the better the agreement between the two tools [32]. We consider the agreement interval, represented by the limits of agreement for energy intake and for macronutrients, sufficiently narrow to support the use of the FFQ. These agreement intervals were similar to those reported in an Italian study comparing a medium length FFQ (36 items) to 3-DFR [37].

It must be emphasised that one of the difficulties met by the interviewers was to obtain from the participants the diversity and quantity of oil and fat intakes in their diet. Furthermore, participants had a tendency to not report oil and fats used in homemade cooked meals in the 3-DFR. Consequently, it is difficult to determine whether our FFQ over- or underestimated lipid intakes and/or to what level they were underestimated by the participants in the 3-DFR, a limit that has been met in another FFQ validation study [21]. This might explain the equal plot distribution of differences in lipid intakes around the mean of differences (close to zero) between FFQ and 3-DFR shown in the Bland Altman plot. However, the low percentage of subjects misclassified in quartile for lipid-related nutrients (SFA, MUFA, PUFA and cholesterol) and the good agreement assessed by weighted kappa values support the use of the FFQ to rank lipid intakes inside a group.

As stated by Willet & Lenart, reproducibility refers to consistency of questionnaire measurements on more than one administration to the same person at different times, considering that conditions are never identical on repeated administration [29]. Intraclass correlation application assess consistency or reproducibility of measurements and coefficient reflects both degree of correlation and agreement between measurements [35]. While a minimum of 3 raters and a sample size of 30 have been suggested for reliability studies [35], the intraclass correlation coefficients obtained in our analyses were found moderate to good despite having two interviewers and a sample of 29 individuals. As stated by Cade et al., correlation coefficients of 0.5 to 0.7 between two administrations of an FFQ are common [36]. Our results surpassed 0.7 in most of the nutrients tested even though 1 year on average separated the administration of the two FFQs. It has been shown that repeating FFQ administration within 1 month leads to higher correlation coefficients than repeated administrations further apart [36]. In a Finnish study, reproducibility analysis of a 110-item FFQ tested as part as a breast cancer study led to intraclass correlation coefficients between 0.49 and 0.73 [52]. Similar results were found in a 112-item FFQ tested among Lebanese children (0.31 to 0.73) [30]. Furthermore, using quartile classification, we found that the percentage of participants misclassified was below 15% for most nutrients (with the exception of lipids and vitamins A and E) and for Mediterranean score. Further, the weighted kappa values showed moderate to good agreement for all nutrients and Mediterranean score. These results are comparable to a Lebanese study performed in children, although they have reported higher percentage of agreement in quartile classification [30].

Limits were identified to ensure proper usage of the validated FFQ and to avoid misleading conclusions. Accordingly, our FFQ should be utilized to rank individuals as per nutrient intakes rather than to assess their absolute values. It is recognized that the 3-DFR is not a perfect tool to measure dietary intake [53], but there is no gold standard to validate FFQs [53]. One of the constraints of our study was that, to facilitate the analyses, we used two different calculation tools to evaluate nutrient intakes derived from the questionnaire and the food records. However, both tools were developed for a French-Canadian population using nutrient values derived from the 2010 Canadian Nutrient File. Another limitation was that teenagers constituted half the sample used for the FFQ validation. In general, the FFQ was administered to the adolescent with the help of the accompanying parent. When the precise information was difficult to obtain from the adolescent, parents were often able to complete the missing data for main meals. However, parents were not necessarily aware of what was eaten by their children outside the house or as snacks, which constitutes a possible source of imprecision. As stated previously, frequency and quantity of lipid intakes were difficult to capture using the FFQ, which could potentially under- or overestimates them. In addition, we observed that girls had a tendency to underestimate their intakes in the 3-DFR or/and to overestimate them in the FFQ. It has been previously shown in the literature that girls and women have a tendency to restrict their diet when using food records [54]. This limit has to be considered when analyzing data obtained in female participants.

The efforts invested to tailor the FFQ to our targeted population and the complementary nature of analyses completed to ensure the validity and reproducibility of the FFQ represent the main strengths of our study [51]. Few FFQs related to the Mediterranean diet and adapted to French-Canadian [21, 55] and European [56, 57] populations have been published in recent years. Other FFQs have targeted only the specific properties of food (e.g. antioxidant-rich foods) for North American [24, 58], European [13, 59] and Asian [60] populations. Also, while FFQs validated for populations at risk of cardiometabolic diseases were mostly developed in adult populations [24, 55, 57, 59], our FFQ was adapted to adolescents and young adults.

## Conclusion

In conclusion, our results support the reproducibility and accuracy of the developed FFQ to correctly rank individuals according to their dietary intake. The validated FFQ represents a valuable tool for future studies measuring the impact of nutrition in the development of long-term cardiometabolic and bone complications in cALL survivors from the Province of Quebec. This FFQ could further be adapted and validated for other adolescent and young adult French-speaking populations at risk of developing chronic diseases.

## Additional files

Additional file 1: **Table S1.** Food frequency questionnaires and 3-day food records: Comparison of nutrient median (inter-quartile range) daily intake and correlation coefficients in adults, **Table S2.** Food frequency questionnaires and 3-day food records: Comparison of nutrient median (inter-quartile range) daily intake and correlation coefficients in adolescents, **Table S3.** Food frequency questionnaires and 3-day food records: Comparison of nutrient median (inter-quartile range) daily intake and correlation coefficients in males, **Table S4.** Food frequency questionnaires and 3-day food records: Comparison of nutrient median (inter-quartile range) daily intake and correlation coefficients in females, **Table S5.** Food frequency questionnaires and 3-day food records: Comparison of nutrient median (inter-quartile range) daily intake and correlation coefficients in boys, **Table S6.** Food frequency questionnaires and 3-day food records: Comparison of nutrient median (inter-quartile range) daily intake and correlation coefficients in men, **Table S7.** Food frequency questionnaires and 3-day food records: Comparison of nutrient median (inter-quartile range) daily intake and correlation coefficients in girls, **Table S8.** Food frequency questionnaires and 3-day food records: Comparison of nutrient median (inter-quartile range) daily intake and correlation coefficients in women, **Table S9.** Food frequency questionnaires and 3-day food records: Comparison of nutrient mean daily intake and correlation coefficients, **Table S10.** Food frequency questionnaires at visits 1 and 2: comparison of nutrient mean daily intake and intraclass correlation coefficients. (DOCX 69 kb)

Additional file 2: **Figure S1.** Bland-Altman plots showing agreement between the average FFQ at visit 1 and 2 for (a) carbohydrates, (b) proteins, (c) lipids, (d) energy intake, (e) vitamin D and (f) calcium (g) vitamin A, (h) vitamin C, (i) vitamin E. A mean of 0 indicates that the 2 tools are in perfect agreement. LOA, limit of agreement; IU, international unit. (PPTX 160 kb)

## Abbreviations

3-DFR: 3-day food record
ALL: Acute lymphoblastic leukemia
cALL: childhood acute lymphoblastic leukemia
CHO: Carbohydrates
CNF: Canadian Nutrient File
FFQ: Food frequency questionnaire
MetS: Metabolic syndrome
MUFA: Monounsaturated fatty acids
PUFA: Polyunsaturated fatty acids
SFA: Saturated fatty acids
