# Supplementary material for: Development and relative validation of a food frequency questionnaire for French-Canadian adolescent and young adult survivors of acute lymphoblastic leukemia
Source: Nutr J. 2018 Apr 21;17:45. doi: 10.1186/s12937-018-0355-9 (PMC5911374; doi:10.1186/s12937-018-0355-9)
Supplement: Supplementary file 1 — Table S1. Food frequency questionnaires and 3-day food records: Comparison of nutrient median (inter-quartile range) daily intake and correlation coefficients in adults, Table S2. Food frequency questionnaires and 3-day food records: Comparison of nutrient median (inter-quartile range) daily intake and correlation coefficients in adolescents, Table S3. Food frequency questionnaires and 3-day food records: Comparison of nutrient median (inter-quartile range) daily intake and correlation coefficients in males, Table S4. Food frequency questionnaires and 3-day food records: Comparison of nutrient median (inter-quartile range) daily intake and correlation coefficients in females, Table S5. Food frequency questionnaires and 3-day food records: Comparison of nutrient median (inter-quartile range) daily intake and correlation coefficients in boys, Table S6. Food frequency questionnaires and 3-day food records: Comparison of nutrient median (inter-quartile range) daily intake and correlation coefficients in men, Table S7. Food frequency questionnaires and 3-day food records: Comparison of nutrient median (inter-quartile range) daily intake and correlation coefficients in girls, Table S8. Food frequency questionnaires and 3-day food records: Comparison of nutrient median (inter-quartile range) daily intake and correlation coefficients in women, Table S9. Food frequency questionnaires and 3-day food records: Comparison of nutrient mean daily intake and correlation coefficients, Table S10. Food frequency questionnaires at visits 1 and 2: comparison of nutrient mean daily intake and intraclass correlation coefficients. (DOCX 69 kb) [file 12937_2018_355_MOESM1_ESM.docx]

**Table S1:** Food frequency questionnaires and 3-day food records: Comparison of nutrient median (inter-quartile range) daily intake and correlation coefficients in adults

|  | **Median daily intake** | | | **Correlations^3^** | |
| --- | --- | --- | --- | --- | --- |
|  | **3-DFR** | **FFQ^1^** | **Difference (%)^2^** | **Unadjusted** | **Energy-adjusted^4^** |
| Energy (kcal) | 2334 (1846 – 2685) | 2532 (2076 – 2940)*** | 11.8 (-1.0 – 23.3) | 0.76** |  |
| Protein (g) | 96.1 (82.1 – 119) | 109 (83.4 – 131)** | 7.1 (-3.7 – 30.9) | 0.66** | 0.46** |
| CHO (g) | 257 (214 – 318) | 318 (253 – 385)*** | 22.6 (-1.2 – 49.2) | 0.59** | 0.30 |
| Lipids (g) | 87.0 (67.6 – 107) | 86.2 (75.5 – 106) | 1.1 (-13.3 – 18.4) | 0.73** | 0.18 |
| SFA (g) | 29.0 (19.6 – 39.6) | 29.0 (23.7 – 36.5) | -3.3 (-20.0 – 21.4) | 0.76** | 0.35* |
| MUFA (g) | 30.7 (24.4 – 40.2) | 31.1 (26.7 – 38.6) | 1.6 (-14.2 – 25.4) | 0.59** | 0.24 |
| PUFA (g) | 17.6 (11.8 – 20.3) | 15.9 (13.2 – 18.9) | -0.2 (-11.9 – 24.3) | 0.48** | 0.14 |
| Cholesterol (mg) | 308 (219 – 402) | 271 (196 – 373) | -10.9 (-34.1 – 29.2) | 0.49** | 0.29 |
| Dietary fiber (g) | 19.1 (15.7 – 24.0) | 25.3 (19.9 – 31.0)*** | 27.0 (-0.8 – 54.3) | 0.43** | 0.54** |
| Alcohol (g) | 3.3 (0.0 – 8.8) | 8.2 (1.4 – 12.8)* | -18.8 (-52.2 – 172) | 0.39* | 0.49** |
| Vitamin A (μg) | 738 (592 – 1029) | 979 (822 – 1232)** | 40.4 (-7.0 – 76.3) | 0.44** | 0.33* |
| Vitamin C (mg) | 122 76.9 – 155) | 191 (143 – 273)*** | 53.6 (11.1 – 134) | 0.47** | 0.39* |
| Vitamin E (mg) | 6.5 (4.8 – 9.0) | 9.8 (7.9 – 10.7)*** | 57.5 (20.9 – 82.7) | 0.55** | 0.53** |
| Vitamin D (IU) | 169 (114 – 244) | 223 (149 – 313)** | 38.7 (-18.6 – 81.1) | 0.63** | 0.49** |
| Calcium (mg) | 1062 (648 – 1425) | 1311 (994 – 1734) *** | 30.2 (3.6 – 55.0) | 0.71** | 0.45** |
| Iron (mg) | 13.7 (11.6 – 18.2) | 16.8 (13.3 – 21.9)** | 13.4 (2.0 – 42.2) | 0.52** | 0.28 |
| Folate (mcg) | 404 (307 – 469) | 530 (430 – 687)*** | 45.0 (17.9 – 69.4) | 0.44** | 0.41** |

Values are medians (IQR). N = 40 adults. ^1^ Wilcoxon signed-rank tests were used to compare values obtained from both FFQ and 3-DFR.^2^ (Daily intake as per FFQ – Daily intake as per 3-DFR)/(Daily intake as per 3-DFR) x 100. ^3^ Spearman correlations were used to assess the relationship of energy, nutrient intakes and energy-adjusted nutrient intakes between 3-DFR and FFQ. ^4^ The residual method was used to calculate the energy-adjusted variables. *P ≤ 0.05; **P ≤ 0.01; ***P ≤ 0.0001

**Table S2:** Food frequency questionnaires and 3-day food records: Comparison of nutrient median (inter-quartile range) daily intake and correlation coefficients in adolescents

|  | **Median daily intake** | | | **Correlations^3^** | |
| --- | --- | --- | --- | --- | --- |
|  | **3-DFR** | **FFQ^1^** | **Difference (%)^2^** | **Unadjusted** | **Energy-adjusted^4^** |
| Energy (kcal) | 1971 (1860 – 2280) | 2213 (1997 – 2693)** | 12.3 (-1.1 – 27.3) | 0.62** |  |
| Protein (g) | 85.6 (73.1 – 100) | 98.7 (81.9 – 119)** | 18.8 (0.6 – 35.2) | 0.45** | 0.53** |
| CHO | 244 (217 – 277) | 285 (255 – 349)*** | 18.9 (4.8 – 34.7) | 0.41** | 0.38* |
| Lipids (g) | 82.2 (68.3 – 98.1) | 83.3 (73.2 – 93.1) | 3.4 (-12.4 – 14.7) | 0.73** | 0.39* |
| SFA (g) | 29.1 (23.4 – 37.1) | 27.7 (24.0 – 30.6) | -7.7 (-17.9 – 9.3) | 0.61** | 0.31 |
| MUFA (g) | 27.4 (22.3 – 33.1) | 29.1 (25.1 – 34.1) | 6.6 (-13.3 – 27.4) | 0.58** | 0.30 |
| PUFA (g) | 13.7 (10.4 – 17.2) | 15.6 (12.7 – 19.7) | 17.3 (-9.1 – 29.5) | 0.67** | 0.54** |
| Cholesterol (mg) | 238 (179 – 331) | 246 (197 – 298) | -3.1 (-24.0 – 32.9) | 0.40* | 0.16 |
| Dietary fiber | 15.3 (12.8 – 18.2) | 23.1 (16.6 – 28.4)*** | 35.1 (9.9 – 67.8) | 0.52** | 0.63** |
| Alcohol (g) | 0.0 (0.0 – 0.0) | 0.0 (0.0 – 0.0)* | -32.2 (-94.7 – 898) | 0.30 | 0.20 |
| Vitamin A (μg) | 705 (548 – 949) | 920 (703 – 1166)** | 18.2 (-8.7 – 80.4) | 0.50** | 0.52** |
| Vitamin C | 122 (68.3 – 196) | 183 (133 – 244)*** | 47.2 (16.2 – 117) | 0.60** | 0.61** |
| Vitamin E (mg) | 5.9 (4.5 – 8.1) | 8.4 (7.1 – 10.3)*** | 44.2 (15.2 – 68.3) | 0.48** | 0.43** |
| Vitamin D (IU) | 178 (113 – 276) | 226 (162 – 283)* | 19.0 (-12.3 – 83.9) | 0.52** | 0.49** |
| Calcium (mg) | 1080 (728 – 1398) | 1303 (1037 – 1473)*** | 21.5 (0.7 – 46.2) | 0.66** | 0.59** |
| Iron (mg) | 11.8 (10.4 – 14.5) | 16.0 (13.2 – 18.1)*** | 19.2 (4.3 – 54.2) | 0.38* | 0.54** |
| Folate (mcg) | 324 (280 – 407) | 480 (374 – 577)*** | 35.3 (13.7 – 78.4) | 0.44** | 0.40* |

Values are medians (IQR). N = 40 adolescents. ^2^ Wilcoxon signed-rank tests were used to compare values obtained from both FFQ and 3-DFR. ^1^ (Daily intake as per FFQ – Daily intake as per 3-DFR)/(Daily intake as per 3-DFR) x 100. ^3^ Spearman correlations were used to assess the relationship of energy, nutrient intakes and energy-adjusted nutrient intakes between 3-DFR and FFQ. ^4^ The residual method was used to calculate the energy-adjusted variables. *P ≤ 0.05; **P ≤ 0.01; ***P ≤ 0.000

**Table S3:** Food frequency questionnaires and 3-day food records: Comparison of nutrient median (inter-quartile range) daily intake and correlation coefficients in males

|  | **Median daily intake** | | | **Correlations^3^** | |
| --- | --- | --- | --- | --- | --- |
|  | **3-DFR** | **FFQ^1^** | **Difference (%)^2^** | **Unadjusted** | **Energy-adjusted^4^** |
| Energy (kcal) | 2384 (2081 – 2917) | 2772 (2386 – 3148) | 14.0 (3.0 – 24.9) | 0.64** |  |
| Protein (g) | 103 (86.6 – 123) | 121 (97.1 – 134) | 11.4 (-4.1 – 37.1) | 0.62** | 0.61** |
| CHO | 267 (234 – 349) | 378 (279 – 413) | 23.0 (4.2 – 46.6) | 0.51** | 0.32* |
| Lipids (g) | 99.5 (81.7 – 119) | 91.9 (83.1 – 114) | 3.4 (-10.9 – 12.4) | 0.64** | 0.16 |
| SFA (g) | 35.6 (27.9 – 45.2) | 30.6 (26.3 – 40.7) | -4.7 (-22.3 – 18.5) | 0.55** | 0.21 |
| MUFA (g) | 33.7 (28.4 – 42.0) | 35.2 (29.3 – 41.4) | 5.3 (-8.4 – 21.6) | 0.64** | 0.41** |
| PUFA (g) | 17.0 (13.3 – 21.1) | 18.4 (15.3 – 20.8) | 5.1 (-5.9 – 25.9) | 0.59** | 0.39* |
| Cholesterol (mg) | 340 (232 – 432) | 298 (237 – 380) | -13.5 (-31.1 – 29.2) | 0.54** | 0.38* |
| Dietary fiber | 16.9 (13.8 – 21.5) | 24.5 (20.5 – 31.0) | 45.3 (3.5 – 68.1) | 0.49** | 0.47** |
| Alcohol (g) | 0.0 (0.0 – 6.6) | 2.3 (0.0 – 10.5) | 0.2 (-56.9 – 383) | 0.64** | 0.53** |
| Vitamin A (μg) | 741 (551 – 1001) | 993 (812 – 1408) | 44.3 (1.0 – 96.5) | 0.56** | 0.35* |
| Vitamin C | 124 (71.0 – 205) | 210 (134 – 279) | 62.0 920.8 – 112) | 0.78** | 0.62** |
| Vitamin E (mg) | 6.9 (4.8 – 9.4) | 10.0 (8.0 – 11.8) | 55.3 (27.1 – 73.5) | 0.55** | 0.46** |
| Vitamin D (IU) | 217 (149 – 325) | 263 (196 – 343) | 35.0 (-16.0 – 88.3) | 0.58** | 0.51** |
| Calcium (mg) | 1256 (931 – 1573) | 1457 (1133 – 1874) | 20.3 (5.5 – 56.2) | 0.76** | 0.59** |
| Iron (mg) | 13.8 (11.7 -17.9) | 18.1 (14.7 -22.2) | 18.2 (5.7 – 44.3) | 0.55** | 0.43** |
| Folate (mcg) | 390 (306 – 479) | 560 (455 – 711) | 43.5 (23.9 – 65.8) | 0.47** | 0.29 |

Values are medians (IQR). N = 40 male. ^1^ Wilcoxon signed-rank tests were used to compare values obtained from both FFQ and 3-DFR. ^2^ (Daily intake as per FFQ – Daily intake as per 3-DFR)/(Daily intake as per 3-DFR) x 100. ^3^ Spearman correlations were used to assess the relationship of energy, nutrient intakes and energy-adjusted nutrient intakes between 3-DFR and FFQ. ^4^ The residual method was used to calculate the energy-adjusted variables. *P ≤ 0.05; **P ≤ 0.01; ***P ≤ 0.0001

**Table S4:** Food frequency questionnaires and 3-day food records: Comparison of nutrient median (inter-quartile range) daily intake and correlation coefficients in females

|  | **Median daily intake** | | | **Correlations^3^** | |
| --- | --- | --- | --- | --- | --- |
|  | **3-DFR** | **FFQ^1^** | **Difference (%)^2^** | **Unadjusted** | **Energy-adjusted^4^** |
| Energy (kcal) | 1885 (1655 – 2119) | 2043 (1771 – 2440) | 11.6 (-3.6 – 24.8) | 0.48** |  |
| Protein (g) | 82.1 (70.3 – 88.6) | 90.6 (75.5 – 106) | 12.7 (-0.2 – 27.9) | 0.37* | 0.31 |
| CHO | 237 (199 – 265) | 268 (240 – 316) | 16.8 (-0.5 – 39.3) | 0.32* | 0.44** |
| Lipids (g) | 73.4 (57.7 – 86.6) | 75.9 (59.3 – 86.2) | -2.0 (-16.8 – 23.1) | 0.61** | 0.40* |
| SFA (g) | 23.5 (18.0 – 31.2) | 24.6 (18.3 – 29.6) | -4.6 (-17.3 – 18.7) | 0.62** | 0.45** |
| MUFA (g) | 24.6 (20.9 – 29.9) | 27.0 (21.7 – 30.1) | -1.7 (-16.8 – 31.4) | 0.28 | 0.04 |
| PUFA (g) | 12.7 (10.1 – 17.9) | 14.0 (11.4 – 16.0) | 1.2 (-14.7 – 29.2) | 0.47** | 0.21 |
| Cholesterol (mg) | 228 (177 – 286) | 208 (182 – 278) | -3.6 (-30.6 – 35.9) | 0.07 | 0.10 |
| Dietary fiber | 17.3 (13.6 – 20.7) | 23.5 (17.0 – 27.5) | 22.7 (-0.8 – 50.9) | 0.53** | 0.61** |
| Alcohol (g) | 0.0 (0.0 – 1.4) | 0.5 (0.0 – 5.9) | -18.8 (-85.7 – 48.2) | 0.49** | 0.40* |
| Vitamin A (μg) | 710 (586 – 960) | 889 (705 – 1007) | 12.7 (-11.8 – 62.9) | 0.41** | 0.41** |
| Vitamin C | 119 (83.5 – 152) | 171 (145 – 239) | 49.7 (3.7 – 130) | 0.20 | 0.23 |
| Vitamin E (mg) | 5.8 (4.4 – 7.4) | 8.4 (6.8 – 10.1) | 41.2 (14.5 – 73.9) | 0.46** | 0.61** |
| Vitamin D (IU) | 138 (99.2 – 221) | 176 (142 – 241) | 19.2 (-14.2 – 77.7) | 0.47** | 0.49** |
| Calcium (mg) | 933 (625 – 1155) | 1085 (880 – 1339) | 30.7 (-3.5 – 45.0) | 0.54** | 0.41** |
| Iron (mg) | 11.8 (10.2 –15.1) | 13.6 (13.0 – 17.2) | 13.9 (-2.8 – 45.6) | 0.27 | 0.32* |
| Folate (mcg) | 326 (270 – 420) | 459 (374 – 537) | 32.3 (11.5 – 76.2) | 0.36* | 0.46** |

Values are medians (IQR). N = 40 female. ^1^ Wilcoxon signed-rank tests were used to compare values obtained from both FFQ and 3-DFR. ^2^ (Daily intake as per FFQ – Daily intake as per 3-DFR)/(Daily intake as per 3-DFR) x 100. ^3^ Spearman correlations were used to assess the relationship of energy, nutrient intakes and energy-adjusted nutrient intakes between 3-DFR and FFQ. ^4^ The residual method was used to calculate the energy-adjusted variables. *P ≤ 0.05; **P ≤ 0.01; ***P ≤ 0.000

**Table S5:** Food frequency questionnaires and 3-day food records: Comparison of nutrient median (inter-quartile range) daily intake and correlation coefficients in boys

|  | **Median daily intake** | | | **Correlations^3^** | |
| --- | --- | --- | --- | --- | --- |
|  | **3-DFR** | **FFQ^1^** | **Difference (%)^2^** | **Unadjusted** | **Energy-adjusted^4^** |
| Energy (kcal) | 2280 (1939 – 2783) | 2693 (2342 – 2957) | 12.3 (1.4 – 27.2) | 0.41 |  |
| Protein (g) | 92.5 (86.0 – 109) | 117 (97.1 – 124) | 19.2 (-2.3 – 39.1) | 0.39 | 0.81** |
| CHO | 268 (224 – 336) | 349 (283 – 399) | 21.8 (7.9 – 39.2) | 0.51* | 0.38 |
| Lipids (g) | 92.2 (82.7 –111) | 91.4 (84.1 – 109) | 3.4 (-10.9 – 9.7) | 0.48* | 0.24 |
| SFA (g) | 37.1 (28.8 – 43.1) | 30.4 (27.2 – 37.4) | -5.1 (-17.4 – 11.2) | 0.37 | 0.02 |
| MUFA (g) | 31.0 (28.3 – 37.0) | 32.9 (29.8 – 39.3) | 6.6 (-5.6 – 20.0) | 0.37 | 0.56* |
| PUFA (g) | 15.6 (13.1 – 20.7) | 18.6 (15.1 – 20.6) | 11.3 (-6.4 – 26.5) | 0.57** | 0.62** |
| Cholesterol (mg) | 309 (222 – 398) | 296 (247 – 336) | -13.5 (-20.6 – 32.0) | 0.49* | 0.44 |
| Dietary fiber | 15.7 (13.2 – 19.4) | 23.1 (16.5 – 30.3) | 46.8 (7.0 – 67.8) | 0.50* | 0.45* |
| Alcohol (g) | 0.0 (0.0 – 0.01) | 0.1 (0.0 – 2.44) | -64.4 (-100 – 1747) | 0.24 | 0.0 |
| Vitamin A (μg) | 717 (551 – 909) | 1005 (783 – 1205) | 39.5 (1.0 – 86.6) | 0.59** | 0.49* |
| Vitamin C | 123.1 (88.8 – 198) | 211 (100 – 251) | 47.2 (20.2 – 100) | 0.81** | 0.68** |
| Vitamin E (mg) | 6.2 (4.7 – 8.5) | 9.4 (7.3 – 11.6) | 52.3 (25.0 – 67.2) | 0.48* | 0.41 |
| Vitamin D (IU) | 241 (167 – 326) | 259 (226 – 372) | 35.0 (-8.8 – 74.7) | 0.54* | 0.57** |
| Calcium (mg) | 1223 (802 – 1599) | 1409 (1145 – 1874) | 22.5 (4.0 – 57.2) | 0.73** | 0.61** |
| Iron (mg) | 12.9 (10.8 – 15.5) | 17.2 (14.1 – 20.3) | 20.6 (5.7 -50.4) | 0.64** | 0.62** |
| Folate (mcg) | 357 (293 – 432) | 541 (401 – 631) | 35.3 (23.5 – 60.5) | 0.48* | 0.35 |

Values are medians (IQR). N = 20 boys. ^1^ Wilcoxon signed-rank tests were used to compare values obtained from both FFQ and 3-DFR. ^2^ (Daily intake as per FFQ – Daily intake as per 3-DFR)/(Daily intake as per 3-DFR) x 100. ^3^ Spearman correlations were used to assess the relationship of energy, nutrient intakes and energy-adjusted nutrient intakes between 3-DFR and FFQ. ^4^ The residual method was used to calculate the energy-adjusted variables. *P ≤ 0.05; **P ≤ 0.01; ***P ≤ 0.0001

**Table S6:** Food frequency questionnaires and 3-day food records: Comparison of nutrient median (inter-quartile range) daily intake and correlation coefficients in men

|  | **Median daily intake** | | | **Correlations^3^** | |  |
| --- | --- | --- | --- | --- | --- | --- |
|  | **3-DFR** | **FFQ^1^** | **Difference (%)^2^** | **Unadjusted** | **Energy-adjusted^4^** |  |
| Energy (kcal) | | 2435 (2251 – 3034) | 2853 (2433 – 3610) | 15.8 (3.7 – 23.0) | 0.80** |  |
| Protein (g) | | 114 (91.6 – 127) | 122 (95.1 – 162) | 3.9 (-4.1 – 33.7) | 0.74** | 0.54* |
| CHO | | 266 (235 – 356) | 385 (273 – 433) | 24.6 (-0.1 – 53.6) | 0.56* | 0.25 |
| Lipids (g) | | 104 (78.8 – 128) | 97.4 (77.2 – 130) | 4.2 (-11.1 – 17.5) | 0.71** | 0.05 |
| SFA (g) | | 34.1 (27.5 – 48.3) | 32.1 (25.0 – 43.2) | -4.6 (-26.4 – 18.5) | 0.73** | 0.25 |
| MUFA (g) | | 40.2 (29.0 – 44.4) | 36.9 (26.8 – 45.8) | 4.0 (-15.9 – 21.7) | 0.69** | 0.35 |
| PUFA (g) | | 17.8 (13.6 – 22.8) | 18.0 (15.3 – 22.7) | 3.2 (-5.8 – 24.6) | 0.58** | 0.21 |
| Cholesterol (mg) | | 345 (264 – 471) | 349 (226 – 473) | -12.0 (-34.1 – 28.3) | 0.62** | 0.27 |
| Dietary fiber | | 18.4 (15.4 – 25.3) | 25.7 (21.9 – 31.9) | 40.5 (3.5 – 68.8) | 0.49* | 0.47* |
| Alcohol (g) | | 5.0 (0.0 – 11.4) | 10.0 (2.3 – 12.4) | 22.5 (-49.2 – 203) | 0.39 | 0.50* |
| Vitamin A (μg) | | 768 (524 – 1167) | 993 (865 – 1753) | 53 (7.1 – 111) | 0.51* | 0.14 |
| Vitamin C | | 123.6 (70.6 – 224.2) | 206 (141 – 294) | 80.4 (29.8 – 179) | 0.73** | 0.57** |
| Vitamin E (mg) | | 7.4 (4.8 – 10.7) | 10.1 (9.3 – 12.9) | 58.1 (27.1 – 95.7) | 0.62** | 0.49* |
| Vitamin D (IU) | | 192 (144 – 293) | 262.9 (174 – 340) | 38.7 (-20.3 – 89.2) | 0.59** | 0.34 |
| Calcium (mg) | | 1286 (939 – 1573) | 1532 (1111 – 1910) | 18.4 (6.9 – 48.6) | 0.76** | 0.61** |
| Iron (mg) | | 13.8 (12.3 – 20.8) | 18.7 (14.9 – 22.9) | 14.0 (3.2 – 37.9) | 0.42 | 0.23 |
| Folate (mcg) | | 409 (318 – 524) | 600 (470 – 743) | 49.7 (24.6 – 72.5) | 0.42 | 0.24 |

Values are medians (IQR). N = 20 men. ^1^ Wilcoxon signed-rank tests were used to compare values obtained from both FFQ and 3-DFR.^2^ (Daily intake as per FFQ – Daily intake as per 3-DFR)/(Daily intake as per 3-DFR) x 100. ^3^ Spearman correlations were used to assess the relationship of energy, nutrient intakes and energy-adjusted nutrient intakes between 3-DFR and FFQ. ^4^ The residual method was used to calculate the energy-adjusted variables. *P ≤ 0.05; **P ≤ 0.01; ***P ≤ 0.000

**Table S7**: Food frequency questionnaires and 3-day food records: Comparison of nutrient median (inter-quartile range) daily intake and correlation coefficients in girls

|  | **Median daily intake** | | | **Correlations^3^** | |
| --- | --- | --- | --- | --- | --- |
|  | **3-DFR** | **FFQ^1^** | **Difference (%)^2^** | **Unadjusted** | **Energy-adjusted^4^** |
| Energy (kcal) | 1875 (1647 – 2060) | 1997 (1771 – 2190) | 12.1 (-3.0 – 27.5) | 0.12 |  |
| Protein (g) | 75.1 (68.1 – 85.3) | 84.2 (73.3 – 99.8) | 16.8 (0.6 – 27.9) | 0.07 | 0.18 |
| CHO | 239 (206 – 254) | 265 (244 – 292) | 14.1 (1.5 – 33.5) | -0.15 | 0.32 |
| Lipids (g) | 68.3 (57.7 – 78.0) | 74.4 (52.0 – 81.2) | 3.6 (-17.3 -23.1) | 0.57** | 0.55* |
| SFA (g) | 23.5 (19.7 – 29.1) | 24.0 (15.1 – 29.0) | -11.9 (-18.5 – 9.3) | 0.47* | 0.49* |
| MUFA (g) | 22.3 (18.1 – 25.5) | 25.2 (20.0 – 28.0) | 4.9 (-22.2 – 31.4) | 0.12 | 0.04 |
| PUFA (g) | 11.3 (9.3 – 14.8) | 13.3 (11.1 – 15.9) | 20.4 (-9.7 – 32.9) | 0.67** | 0.44 |
| Cholesterol (mg) | 197 (176 – 248) | 203 (183 – 236) | 0.8 (-30.6 – 40.9) | -0.22 | -0.20 |
| Dietary fiber | 14.5 (12.4 – 17.7) | 22.1 (16.6 – 24.3) | 27.8 (9.9 – 68.0) | 0.55* | 0.67** |
| Alcohol (g) | 0.0 (0.0 – 0.0) | 0.0 (0.0 – 0.0) | 0.0 (-89.4 – 50.0) | 0.45* | 0.41 |
| Vitamin A (μg) | 705 (520 – 1044) | 856 (641 – 1007) | 3.5 (-12.4 – 72.2) | 0.44 | 0.54* |
| Vitamin C | 119.6 (67.1 – 192.8) | 170 (149 – 234) | 46.7 (2.2 – 159) | 0.37 | 0.42 |
| Vitamin E (mg) | 5.3 (4.2 – 7.7) | 8.0 (6.9 – 8.8) | 37.1 (14.5 – 72.9) | 0.31 | 0.48* |
| Vitamin D (IU) | 138 (99.0 – 225) | 166 (152 – 224) | 13.1 (-19.5 – 83.9) | 0.11 | 0.32 |
| Calcium (mg) | 961 (626 – 1139) | 1063 (887 – 1317) | 20.9 (-3.7 – 39.8) | 0.49* | 0.49* |
| Iron (mg) | 11.6 (10.2 – 12.8) | 13.4 (13.0 – 16.3) | 16.5 (-4.4 – 57.0) | -0.12 | 0.29 |
| Folate (mcg) | 321 (257 – 381) | 450 (371 – 485) | 32.0 (11.5 – 85.6) | 0.21 | 0.42 |

Values are medians (IQR). N = 20 girls. ^1^ Wilcoxon signed-rank tests were used to compare values obtained from both FFQ and 3-DFR. ^2^ (Daily intake as per FFQ – Daily intake as per 3-DFR)/(Daily intake as per 3-DFR) x 100. ^3^ Spearman correlations were used to assess the relationship of energy, nutrient intakes and energy-adjusted nutrient intakes between 3-DFR and FFQ. ^4^ The residual method was used to calculate the energy-adjusted variables. *P ≤ 0.05; **P ≤ 0.01; ***P ≤ 0.0001

**Table S8:** Food frequency questionnaires and 3-day food records: Comparison of nutrient median (inter-quartile range) daily intake and correlation coefficients in women

|  | **Median daily intake** | | | **Correlations^3^** | |
| --- | --- | --- | --- | --- | --- |
|  | **3-DFR** | **FFQ^1^** | **Difference (%)^2^** | **Unadjusted** | **Energy-adjusted^4^** |
| Energy (kcal) | 1928 (1659 – 2358) | 2250 (1761 – 2542) | 10.0 (-3.6 – 23.3) | 0.61** |  |
| Protein (g) | 85.2 (77.8 – 105) | 98.1 (78.2 – 112) | 9.0 (-0.2 – 27.6) | 0.48* | 0.32 |
| CHO | 225 (191 – 294) | 283 (210.7 – 322) | 20.7 (-1.5 – 46.1) | 0.56** | 0.30 |
| Lipids (g) | 80.3 (58.1 – 92.6) | 79.7 (66.8 -87.7) | -5.6 (-14.4 – 21.7) | 0.52* | 0.22 |
| SFA (g) | 23.5 (16.4 – 31.5) | 25.8 (20.5 – 30.2) | 5.1 (-11.9 – 27.6) | 0.76** | 0.48* |
| MUFA (g) | 29.6 (23.4 – 31.3) | 27.9 (26.0 – 31.6) | -5.3 (-11.3 – 30.3) | 0.12 | 0.10 |
| PUFA (g) | 15.0 (11.1 – 19.1) | 14.7 (11.4 – 16.1) | -6.7 (-34.3 – 16.4) | 0.22 | 0.06 |
| Cholesterol (mg) | 258 (189 – 332) | 238 (175 – 286) | -9.6 (-31.3 – 35.2) | 0.19 | 0.38 |
| Dietary fiber | 19.5 (16.7 – 22.6) | 24.4 (18.2 – 28.7) | 18.8 (-2.3 – 47.4) | 0.47* | 0.51* |
| Alcohol (g) | 0.6 (0.0 – 5.9) | 5.9 (0.5 – 15.3) | -20.2 (-82.0 – 46.4) | 0.27 | 0.46* |
| Vitamin A (μg) | 732 (624 – 809) | 930 (822 – 1021) | 34.7 (-7.5 – 60.1) | 0.36 | 0.20 |
| Vitamin C | 119 (107 – 145) | 176 (144 – 243) | 52.6 (3.7 – 100) | 0.08 | -0.09 |
| Vitamin E (mg) | 6.1 (4.7 – 7.4) | 8.6 (6.8 – 10.5) | 53.4 (10.8 – 74.8) | 0.44 | 0.72** |
| Vitamin D (IU) | 139 (99.2 – 215) | 183 (136 – 268) | 36.9 (-12.7 – 73.2) | 0.70** | 0.58** |
| Calcium (mg) | 933 (587 – 1156) | 1164 (870 – 1483) | 37.5 (-1.7 – 58.9) | 0.53* | 0.34 |
| Iron (mg) | 13.1 (10.6 – 16.9) | 14.6 (12.9 – 17.9) | 10.6 (-2.4 – 42.7) | 0.47* | 0.27 |
| Folate (mcg) | 388 (287 – 447) | 479 (385 – 577) | 32.3 (9.8 – 67.2) | 0.46* | 0.48* |

Values are medians (IQR). N = 20 women. ^1^ Wilcoxon signed-rank tests were used to compare values obtained from both FFQ and 3-DFR.^2^ (Daily intake as per FFQ – Daily intake as per 3-DFR)/(Daily intake as per 3-DFR) x 100. ^3^ Spearman correlations were used to assess the relationship of energy, nutrient intakes and energy-adjusted nutrient intakes between 3-DFR and FFQ. ^4^ The residual method was used to calculate the energy-adjusted variables. *P ≤ 0.05; **P ≤ 0.01; ***P ≤ 0.0001

**Table S9**: Food frequency questionnaires and 3-day food records: Comparison of nutrient mean daily intake and correlation coefficients

|  | **Mean daily intake** | | | **Correlations^3^** | |
| --- | --- | --- | --- | --- | --- |
|  | **3-DFR** | **FFQ^1^** | **Difference (%)^2^** | **Unadjusted** | **Energy-adjusted^4^** |
| Energy (kcal) | 2221 ± 564 | 2472 ± 667*** | 12.6 ± 18.8 | 0.72** |  |
| Protein (g) | 95.3 ± 26.8 | 106 ± 29.0*** | 14.2 ± 25.0 | 0.59** | 0.47** |
| CHO (g) | 264 ± 76.1 | 315 ± 92.5*** | 22.6 ± 28.7 | 0.50** | 0.39** |
| Lipids (g) | 86.8 ± 27.2 | 87.3 ± 25.8 | 3.7 ± 23.3 | 0.74** | 0.28* |
| SFA (g) | 30.6 ± 11.7 | 29.2 ± 10.3 | 0.4 ± 29.4 | 0.69** | 0.33** |
| MUFA (g) | 30.2 ± 10.1 | 31.1 ± 9.6 | 7.4 ± 29.8 | 0.61** | 0.25* |
| PUFA (g) | 15.9 ± 6.2 | 16.2 ± 5.0 | 10.6 ± 36.5 | 0.58** | 0.33** |
| Cholesterol (mg) | 293 ± 127 | 280 ± 117 | 4.7 ± 43.3 | 0.46** | 0.24* |
| Dietary fiber (g) | 18.2 ± 6.3 | 24.1 ± 8.2*** | 38.0 ± 45.7 | 0. 50** | 0.58** |
| Alcohol (g) | 3.9 ± 10.0 | 5.6 ± 8.8* | NA | 0.59** | 0.47** |
| Vitamin A (μg) | 803 ± 381 | 1034± 428*** | 49.0 ± 74.5% | 0.47** | 0. 41** |
| Vitamin C (mg) | 132 ± 68.2 | 204 ± 99,6*** | 98.9 ± 205% | 0.55** | 0.50** |
| Vitamin E (mg) | 6.9 ± 3.1 | 9.4 ± 3.2*** | 53.7 ± 58.4% | 0.54** | 0.47** |
| Vitamin D (IU) | 199 ± 118 | 246 ± 127** | 60.9 ± 154% | 0.58** | 0.50** |
| Calcium (mg) | 1108 ± 480 | 1357 ± 491*** | 33.1 ± 48.1% | 0.70** | 0.52** |
| Iron (mg) | 14.2 ± 5.0 | 17.0 ± 5.3*** | 25.9 ± 37.4% | 0.49** | 0.42** |
| Folate (mcg) | 373 ± 11 | 528 ± 16*** | 48.2 ± 47.6% | 0.46** | 0.41** |

Values are means ± SD, N = 80. ^1^ Wilcoxon signed-rank tests were used to compare values obtained from both FFQ and 3-DFR. ^2^ (Daily intake as per FFQ – Daily intake as per 3-DFR)/(Daily intake as per 3-DFR) x 100. ^3^ Spearman correlations were used to assess the relationship of energy, nutrient intakes and energy-adjusted nutrient intakes between 3-DFR and FFQ. ^4^ The residual method was used to calculate the energy-adjusted variables. *P ≤ 0.05; **P ≤ 0.01; ***P ≤ 0.0001. 3-DFR: 3-day food record, FFQ: food frequency questionnaire, CHO: carbohydrates, SFA: saturated fatty acids; MUFA: monounsaturated fatty acids, PUFA: polyunsaturated fatty acids. Note: Data presented as medians and inter-quartile ranges are available in Table 3.

**Table S10**: Food frequency questionnaire visits 1 and 2: Comparison of nutrient mean daily intake and intraclass correlation coefficients

|  | **Mean daily intake** | | |  |
| --- | --- | --- | --- | --- |
|  | **FFQ V1** | **FFQ V2^2^** | **Difference (%)^1^** | **Correlations^3^** |
| Energy (kcal) | 2548 ± 697 | 2556 ± 828 | 3.3 ± 22.0 | 0.84*** |
| Protein (g) | 110 ± 28.1 | 114 ± 33.8 | 1.0 ± 22.4 | 0.79*** |
| CHO (g) | 325 ± 95.4 | 312 ± 113 | 9.3 ± 27.2 | 0.81*** |
| Lipids (g) | 87.4 ± 27.6 | 93.1 ± 33.1 | -1.1 ± 29.4 | 0.76*** |
| SFA (g) | 27.7 ± 9.5 | 30.1 ± 12.0 | -3.0 ± 27.1 | 0.81*** |
| MUFA (g) | 32.0 ± 10.7 | 34.7 ± 12.3 | -1.8 ± 31.8 | 0.62** |
| PUFA (g) | 17.1 ± 6.3 | 19.6 ± 9.1 | -2.0 ± 48.8 | 0.56* |
| Cholesterol (mg) | 285 ± 115 | 314± 117 | -5.2 ± 29.3 | 0.83*** |
| Dietary fiber (g) | 23.2 ± 8.8 | 24.6 ± 10.2 | 6.4 ± 58.4 | 0.63** |
| Alcohol (g) | 7.6 ± 8.7 | 7.5 ± 9.1 | 21.9 ± 101.4 | 0.92*** |
| Vitamin A (μg) | 1011± 402 | 1124± 453 | -3.6 ± 33.1 | 0.68** |
| Vitamin C (mg) | 210± 104 | 193 ± 106 | 21.2 ± 76.9 | 0.83*** |
| Vitamin E (mg) | 11.2 ± 6.7 | 11.5 ± 8.7 | 8.7 ± 46.0 | 0.86*** |
| Vitamin D (IU) | 257 ± 133 | 264 ± 113 | -2.1 ± 22.9 | 0.88*** |
| Calcium (mg) | 1337 ± 440 | 1359 ± 427 | 1.8 ± 24.6 | 0.87*** |
| Iron (mg) | 16.4 ± 4.7 | 17.1 ± 6.4 | 2.2 ± 27.7 | 0.76*** |
| Folate (mcg) | 514 ± 153 | 534 ± 180 | 1.2 ± 28.5 | 0.76*** |

Values are means ± SD, N = 29. ^1^(Daily intake as per FFQ V1 – Daily intake as per FFQ V2)/(Daily intake as per FFQ V2) x 100. ^2^Wilcoxon signed-rank tests were used to compare values obtained from both FFQ V1 and FFQ V2. No statistical differences were found. ^3^Intraclass correlations between FFQ V1 and FFQ V2 based on log transformed values. Analyses were completed on crude values not adjusted for energy intake. *P ≤ 0.05; **P ≤ 0.01; ***P ≤ 0.0001. V1, visit 1; V2, visit 2. FFQ: food frequency questionnaire, CHO: carbohydrates, SFA: saturated fatty acids; MUFA: monounsaturated fatty acids, PUFA: polyunsaturated fatty acids. Note: Data presented as medians and inter-quartile ranges are available in Table 5.
