# Supplementary material for: Development and relative validation of a food frequency questionnaire for French-Canadian adolescent and young adult survivors of acute lymphoblastic leukemia
Source: Nutr J. 2018 Apr 21;17:45. doi: 10.1186/s12937-018-0355-9 (PMC5911374; doi:10.1186/s12937-018-0355-9)
Supplement: Supplementary file 2 — Figure S1. Bland-Altman plots showing agreement between the average FFQ at visit 1 and 2 for (a) carbohydrates, (b) proteins, (c) lipids, (d) energy intake, (e) vitamin D and (f) calcium (g) vitamin A, (h) vitamin C, (i) vitamin E. A mean of 0 indicates that the 2 tools are in perfect agreement. LOA, limit of agreement; IU, international unit. (PPTX 160 kb) [file 12937_2018_355_MOESM2_ESM.pptx]

## Slide 1
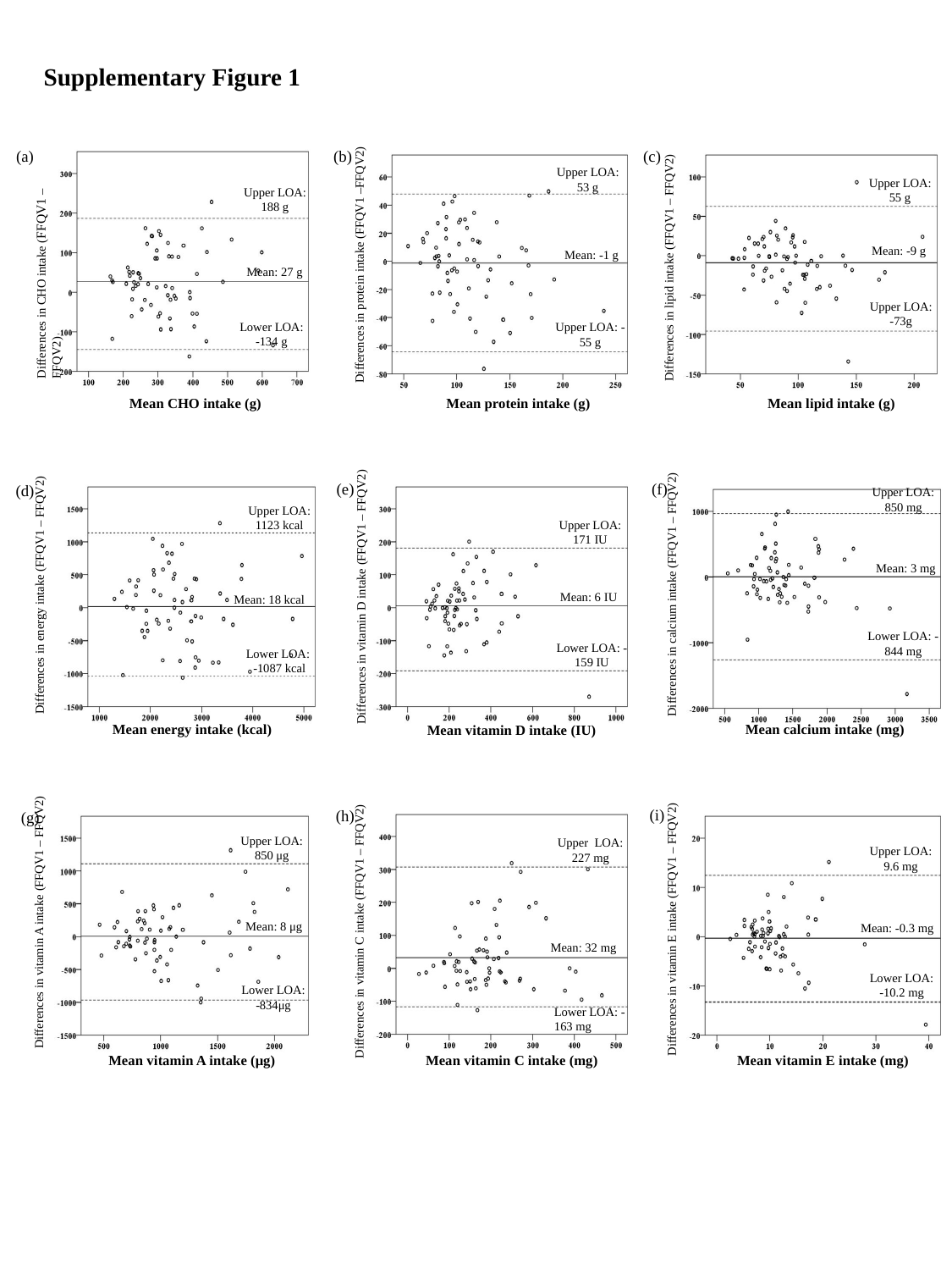

Supplementary Figure 1
Differences in protein intake (FFQV1 –FFQV2)
Differences in CHO intake (FFQV1 – FFQV2)
Differences in lipid intake (FFQV1 – FFQV2)
(a)
(b)
(c)
Upper LOA: 53 g
Upper LOA: 55 g
Upper LOA: 188 g
Mean: -9 g
Mean: -1 g
Mean: 27 g
Upper LOA: -73g
Lower LOA: -134 g
Upper LOA: -55 g
Mean CHO intake (g)
Mean lipid intake (g)
Mean protein intake (g)
Differences in vitamin D intake (FFQV1 – FFQV2)
Differences in calcium intake (FFQV1 – FFQV2)
Differences in energy intake (FFQV1 – FFQV2)
(f)
(e)
(d)
Upper LOA: 850 mg
Upper LOA: 1123 kcal
Upper LOA: 171 IU
Mean: 3 mg
Mean: 6 IU
Mean: 18 kcal
Lower LOA: -844 mg
Lower LOA: -159 IU
Lower LOA:
-1087 kcal
Mean energy intake (kcal)
Mean calcium intake (mg)
Mean vitamin D intake (IU)
Differences in vitamin E intake (FFQV1 – FFQV2)
Differences in vitamin C intake (FFQV1 – FFQV2)
Differences in vitamin A intake (FFQV1 – FFQV2)
(i)
(h)
(g)
Upper LOA: 850 μg
Upper LOA: 227 mg
Upper LOA: 9.6 mg
Mean: 8 μg
Mean: -0.3 mg
Mean: 32 mg
Lower LOA: -10.2 mg
Lower LOA:
-834μg
Lower LOA: -163 mg
Mean vitamin A intake (μg)
Mean vitamin C intake (mg)
Mean vitamin E intake (mg)
